# Supplementary material for: Endonuclease G promotes autophagy by suppressing mTOR signaling and activating the DNA damage response
Source: Nat Commun. 2021 Jan 20;12:476. doi: 10.1038/s41467-020-20780-2 (PMC7817833; doi:10.1038/s41467-020-20780-2)
Supplement: Supplementary file 1 — Supplementary Information [file 41467_2020_20780_MOESM1_ESM.pdf]

Supplementary materials for

Endonuclease G promotes autophagy by suppressing mTOR signaling and activating DNA damage response

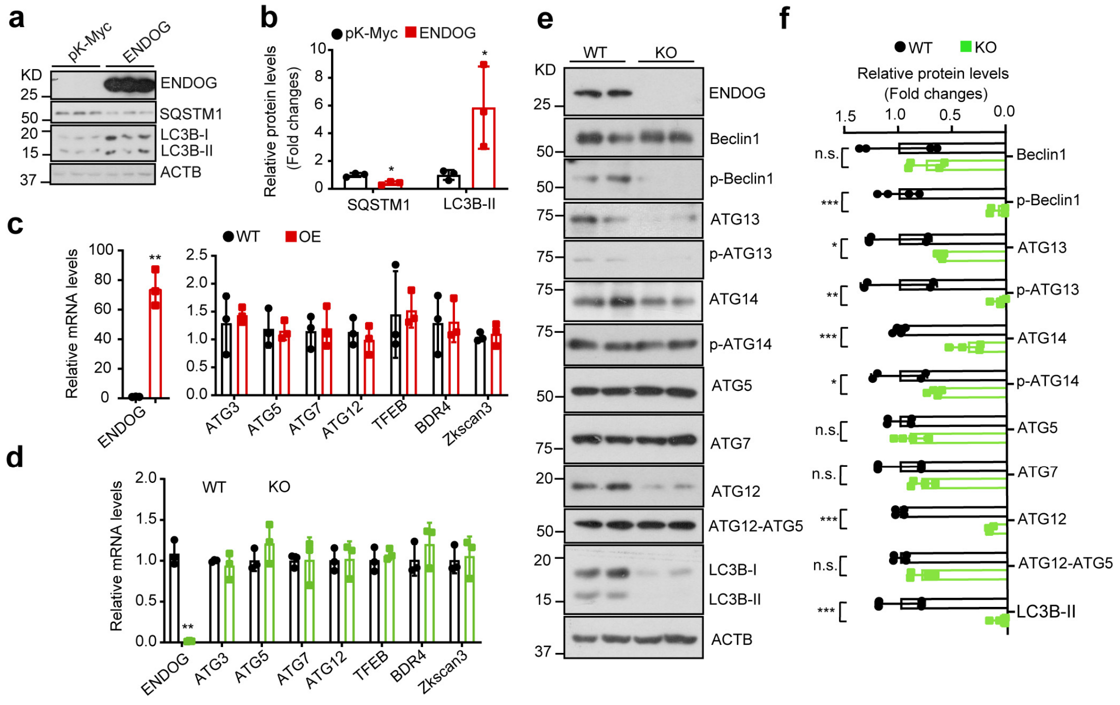

Figure S1. ENDOG promotes autophagy in L02. a-b. Representative western blots and quantitative results of autophagy related proteins (transfected with pK-Myc or ENDOG for 48 hours; n = 3 independent samples; data are presented as mean values ± SD; * $p < 0.05$). **c-d**. qPCR of autophagy related genes in ENDOG knockout (KO) (**c**) or ENDOG overexpressed (OE) (**d**) L02 cells (n = 3 independent samples; data are presented as mean values ± SD; ** $p < 0.01$). **e-f.** Representative western blots and quantitative results of autophagy-related proteinsin wild-type or ENDOG knockout cells. (n = 4 independent samples; data are presented as mean values ± SD; * $p < 0.05$; ** $p < 0.01$, *** $p < 0.001$, n.s. : no significance). Source data are provided as a Source Data file.

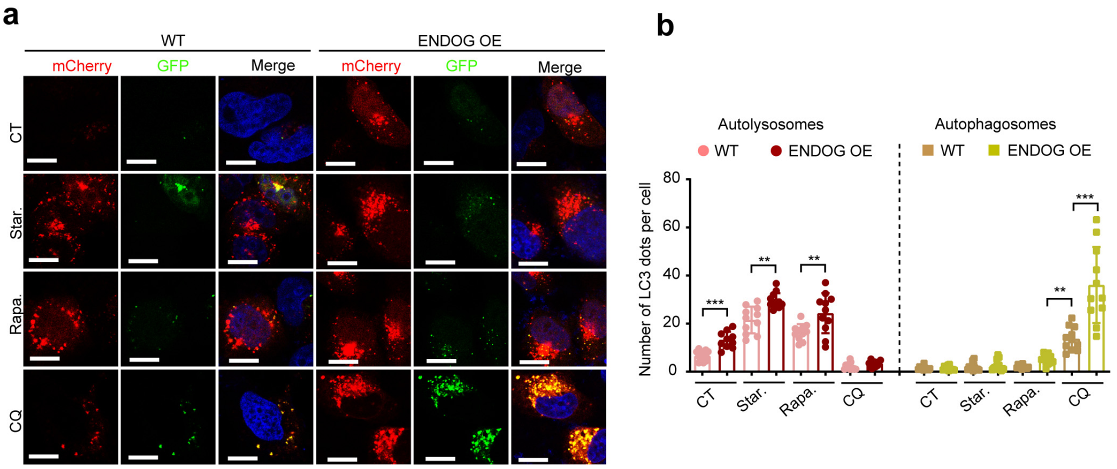

Figure S2. ENDOG promotes autophagic flux in L02 cells. a-b. Representative images (**a**) and quantitative results (**b**) of autophagosome and autolysosome in wild-type and ENDOG overexpressed L02 cells upon different treatments. (WT: wild-type; OE: ENDOG overexpressed; CT: control; Star.: starvation for 6 hours; Rapa.: Rapamycin, 1 μM for 6 hours; CQ: 50 μM for 6 hours; Scale bar =10 μm; n = 100 independent cells exaimined over 3 independent experiments; data are presented as mean values ± SD, ** $p < 0.01$, *** $p < 0.001$). Source data are provided as a Source Data file.

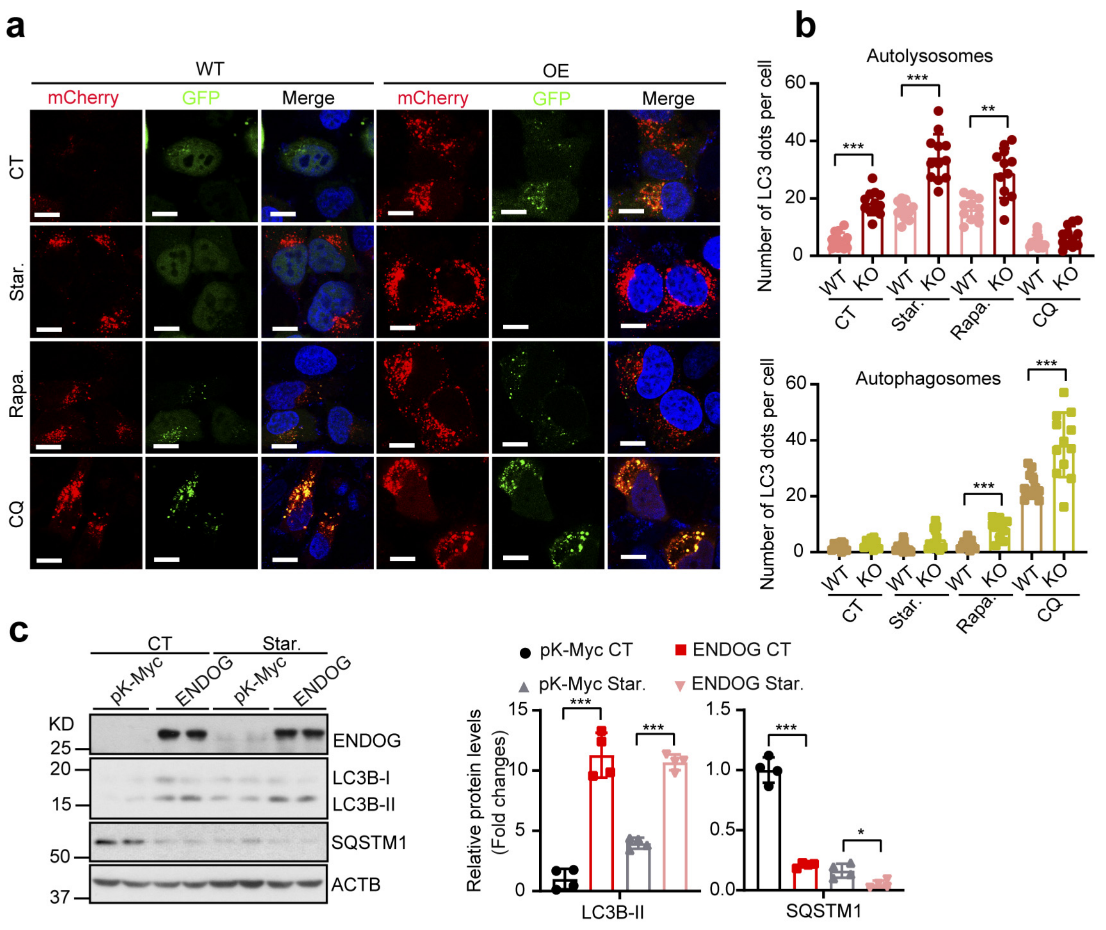

Figure S3. ENDOG promotes autophagic flux in HepG2. a-b. Representative images (**a**) and quantitative results (**b**) of autophagosome and autolysosome in wild-type and ENDOG overexpressed HepG2 cells upon different treatments. (WT: wild-type; OE: ENDOG overexpressed; CT: control; Star.: starvation for 6 hours; Rapa.: Rapamycin, 1μM for 6 hours; CQ: 50 μM for 6 hours; Scale bar =10 μm; n= 100 independent cells exaimined over 3 independent experiments, data are presented as mean values ± SD, ** $p < 0.01$, *** $p < 0.001$). **c.** Representative western blots and quantitative results of autophagy related proteins in HepG2 cells under the normal or starvation conditions. (Star.: starvation for 6 hours;n = 4 independent samples; data are presented as mean values ± SD, * $p < 0.05$; *** $p < 0.001$). Source data are provided as a Source Data file.

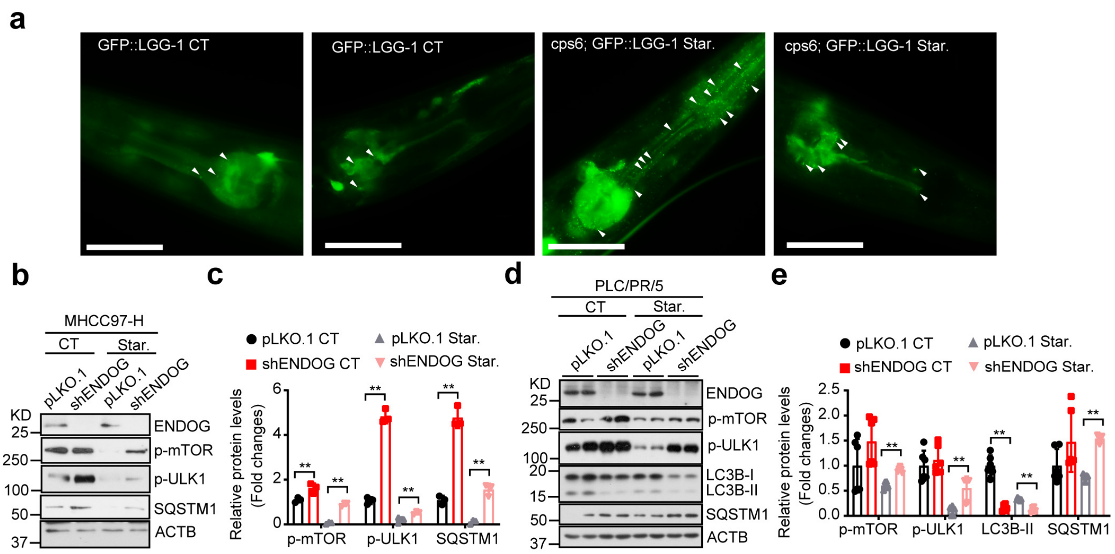

Figure S4. Loss of ENDOG represses starvation-induced autophagy.

a. Representative images of GFP-LGG-1 puncta in the pharyngeal muscle of control or ENDOG loss (*cps-6*;GFP::LGG-1) *C. elegans*. (white arrows, GFP-LGG-1 puncta, Star.: starvation for 4 hours; Scale bar =50 μm; biological repeated three times). **b-e.** Representative western blots andquantitative results of mTOR pathway and autophagy related proteins in MHCC97-H (**b-c**) and PLC/PR/5cells (**d-e**) following the starvation treatment for 6 hours. (n = 3-4 independent samples; data are presented as mean values ± SD, ** P < 0.01). Source data are provided as a Source Data file.

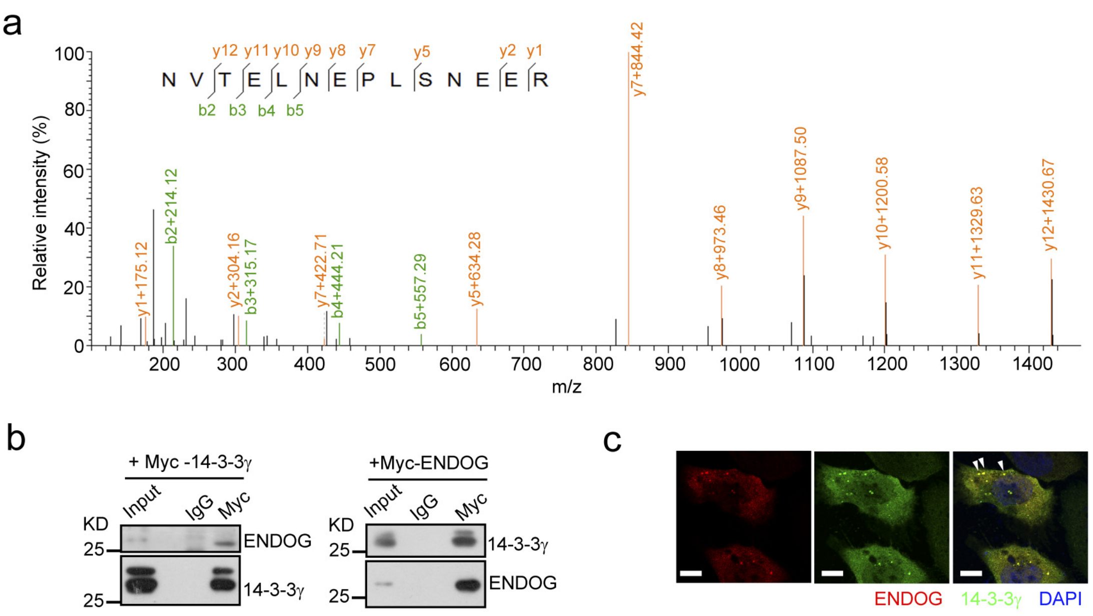

Figure S5. ENDOG interacts with14-3-3γ. a. Mass spectrometry (MS) analysis of 14-3-3γ peptide. **b.** Co-IP experiments showed that interaction between ENDOG and 14-3-3γ in Myc-14-3-3γ (left) or Myc-ENDOG (right) overexpressed L02 cell (biological repeated three times). **c.** Representative IF staining images showed the co-localizationof 14-3-3γ and ENDOG in L02 cell (white arrows). (Scale bar =10 μm; biological repeated three times). Source data are provided as a Source Data file.

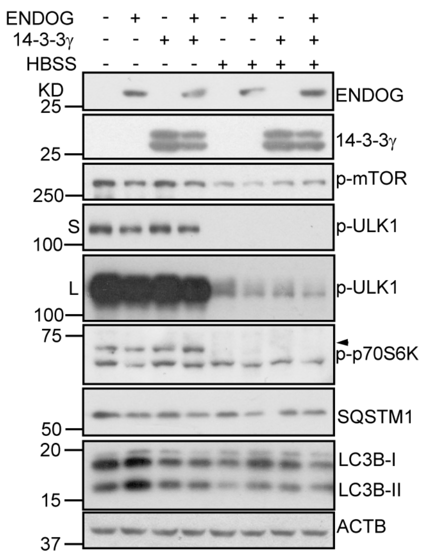

Figure S6. 14-3-3γ repressed the ENDOG-induced mTOR repression and

autophagy. Western blots of the indicated proteins. (ENDOG or 14-3-3γ were transfected for 48 hours; HBSS treated for 6 hours; S: short exposure; L: long exposure; biological repeated three times). Source data are provided as a Source Data file.

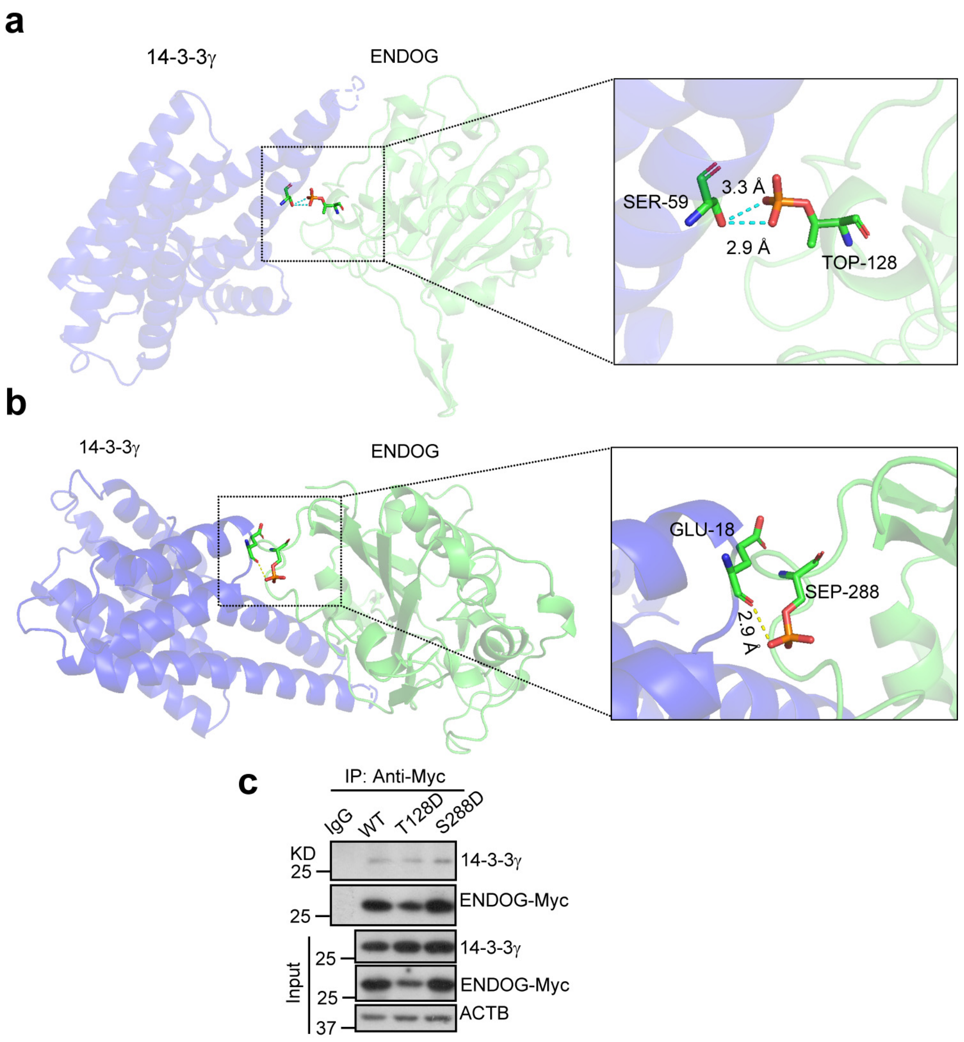

Figure S7. Interactive docking prediction of 14-3-3γ and ENDOG by Zdock Server and Co-IP confirmation. a. The phosphorylated Threonine-128 of ENDOG have two hydrogen bonds with Serine-59 of 14-3-3γ. **b.** The phosphorylated Serine-288 of ENDOG have one hydrogen bonds with Glutamic Acid-18 of 14-3-3γ. **c.** Wild-typeor indicated mutant ENDOG were overexpressed in the ENDOG knockout cells for 48 hours. Co-IP experiments were performed by Anti-myc. **(**WT: wild-type; T128D: T128

to D128 mutation of ENDOG; S288D:S288 to D288 mutation of ENDOG; biological repeated three times). Source data are provided as a Source Data file.

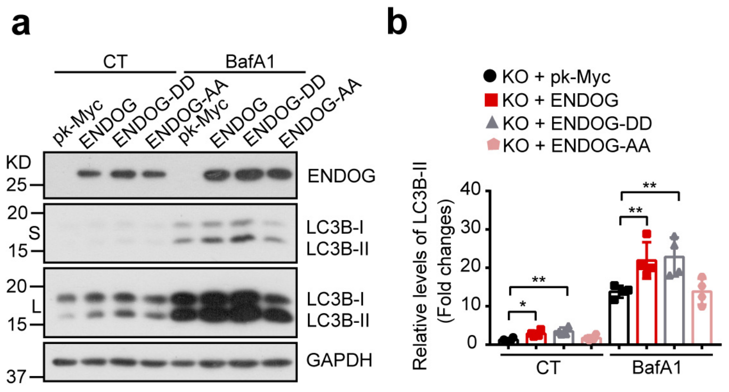

Figure S8. Phosphorylation of T128 and S288 is necessary for ENDOG-mediated autophagy. a-b. Western blots of the indicated proteins (**a**) and the quantification of LCEB-II (**b**). (ENDOG KO cells transfected with pK-Myc, wild type ENDOG, ENDOG-DD and ENDOG-AA for 48 hours; BafA1: 100 nM, for 6 hours; S: short exposure; L: long exposure; n = 4 independent samples; data are presented as mean values ± SD, * $p < 0.05$; ** $p < 0.01$). Source data are provided as a Source Data file.

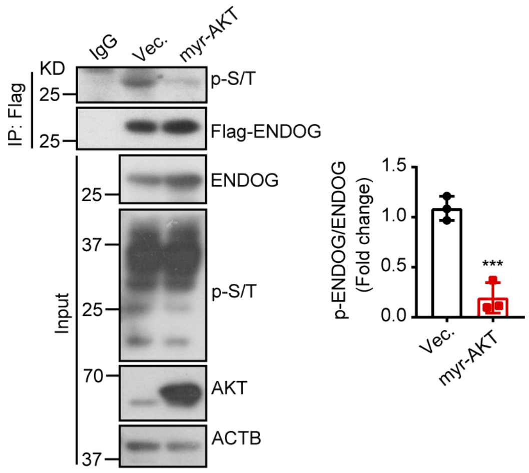

Figure S9. AKT represses the phosphorylation of ENDOG. Overexpression of

activated AKT represses the phosphorylation of ENDOG in ENDOG overexpressed L02, quantitative results on right. (Vec.: empty vector; myr-AKT: constitutively activated AKT; co-overexpressed Flag-ENDOG and myr-AKT/Vec for 48 hours; n = 3 independent experiments; data are presented as mean values ± SD; *** $p < 0.001$). Source data are provided as a Source Data file.

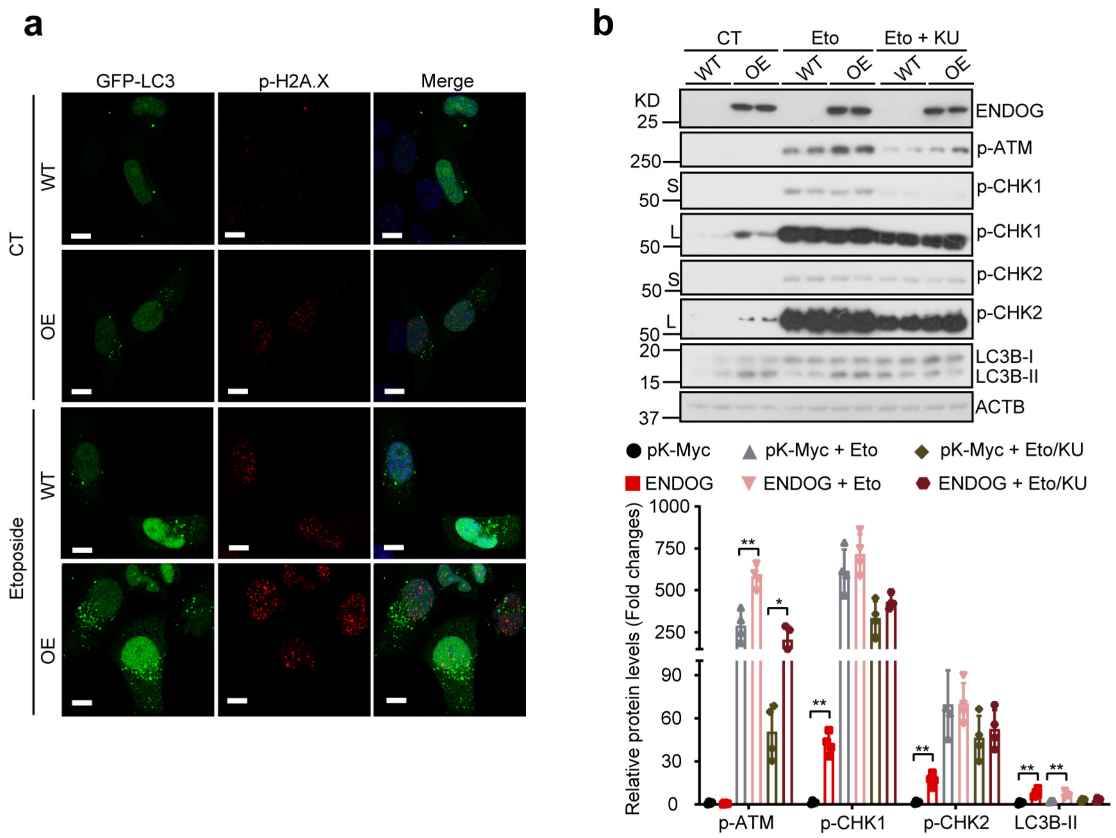

Figure S10. ENDOG promotes autophagy through activating the DNA damage response. a. Representative images of p-H2A.X foci and autophagosome (GFP-LC3 puncta) in wild-type and ENDOG overexpressed cells following the etoposide treatment (Etoposide: 50 μM for 1 hour; Scale bar =10 μm). **b.** Representativewestern blots (upper) andquantitative results (lower) of p-ATM, p-CHK1, p-CHK2, LC3B and ENDOG in wild-type and ENDOG overexpressed cells following the indicated

treatments (WT: wild-type; OE: ENDOG overexpressed; Eto: Etoposide, 50 µM for 1 hour; KU: KU-60019, 10 µM for 1 hour; L: long exposure; S: short exposure; n = 4 independent samples; data are presented as mean values ± SD* $p < 0.05$; ** $p < 0.01$). Source data are provided as a Source Data file.

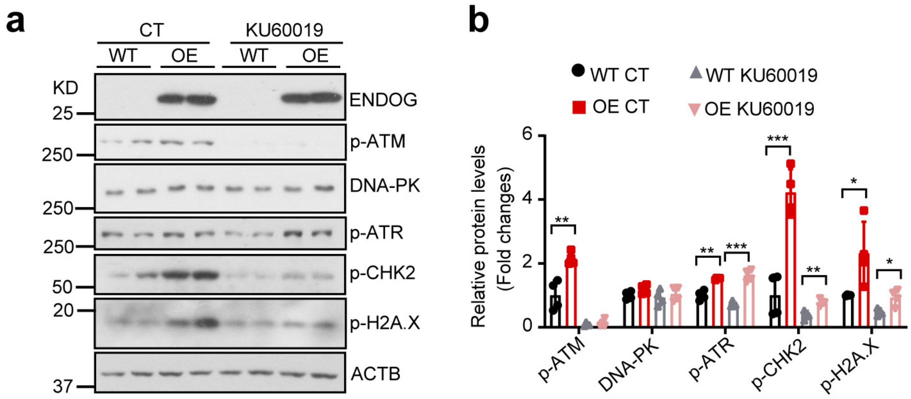

Figure S11. KU60019 treatment partially repressed the ENDOG-induced DNA damage. a-b. Western blots (**a**) and quantification (**b**) of the indicated proteins in wild-type and ENDOG overexpressed cells following the KU60019 treatment. (KU60019: 10 µM for 1 hour; n = 4 independent samples; * $p < 0.05$; ** $p < 0.01$; *** $p < 0.001$). Source data are provided as a Source Data file.

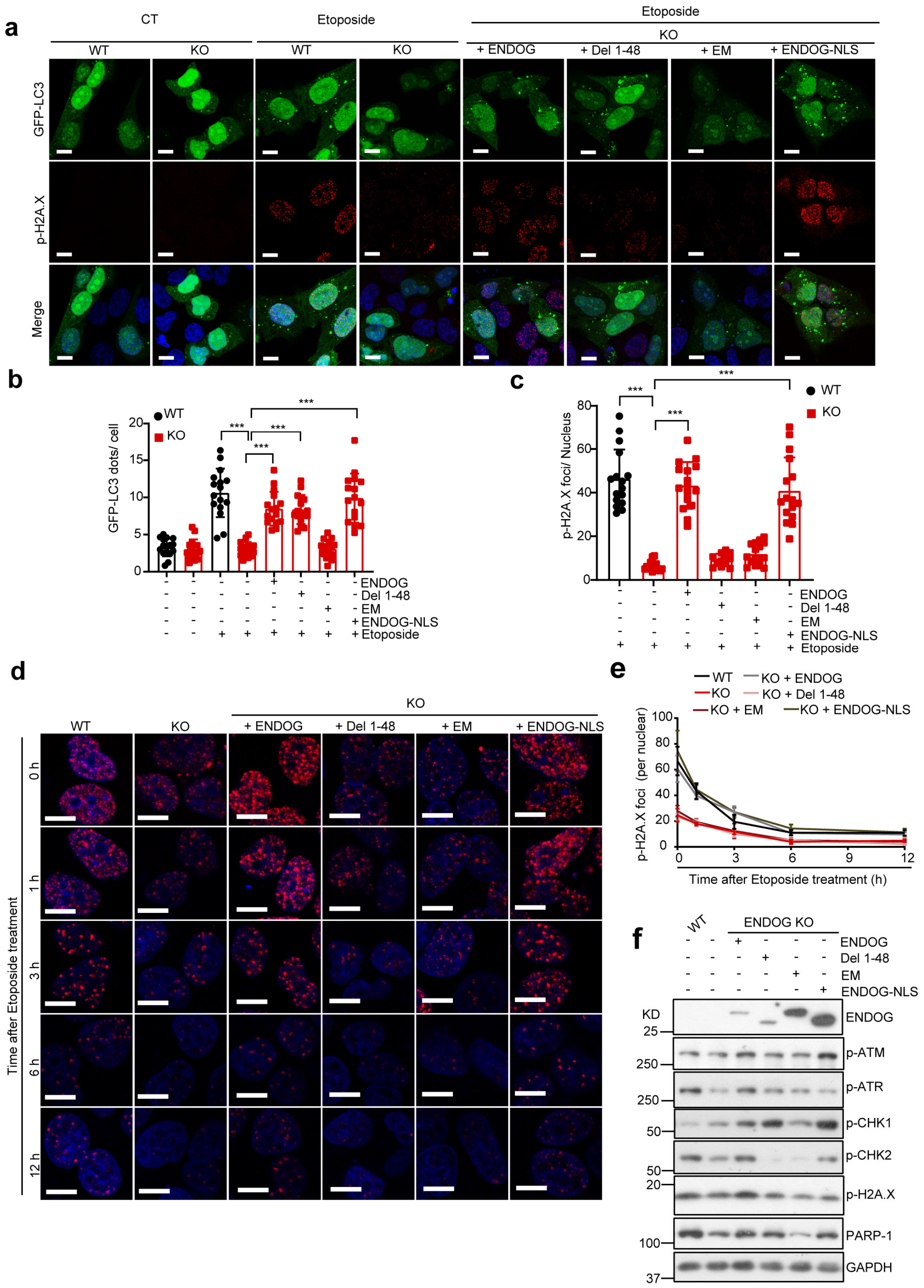

Figure S12. Endonuclease activity of ENDOG is essential for the DNA damage response and autophagy induction. a-c. Representative images (**a**) and respective quantitative results (**b**) of GFP-LC3 puncta and p-H2A.X foci under etoposide

treatment (**c**). (WT: wild-type; KO: ENDOG knockout; plasmids transiently transfected for 48 hours; etoposide: 50 μM for 1 hour; Scale bar = 10 μm; n = 100 independent cells exaimined over 3 independent experiments, data are presented as mean values ± SD, *** $p < 0.001$). **d-e.** Representative images of p-H2A.X foci (**d**) and quantitative results (**e**) in the indicated cell groups at different time points after etoposide treatment (Scale bar = 10 μm; n = 50 independent cells). **f**. Western blots of the indicated proteins in wild-type, ENDOG KO cells, and ENDOG KO cells transfected with the ENDOG mutants. Source data are provided as a Source Data file.

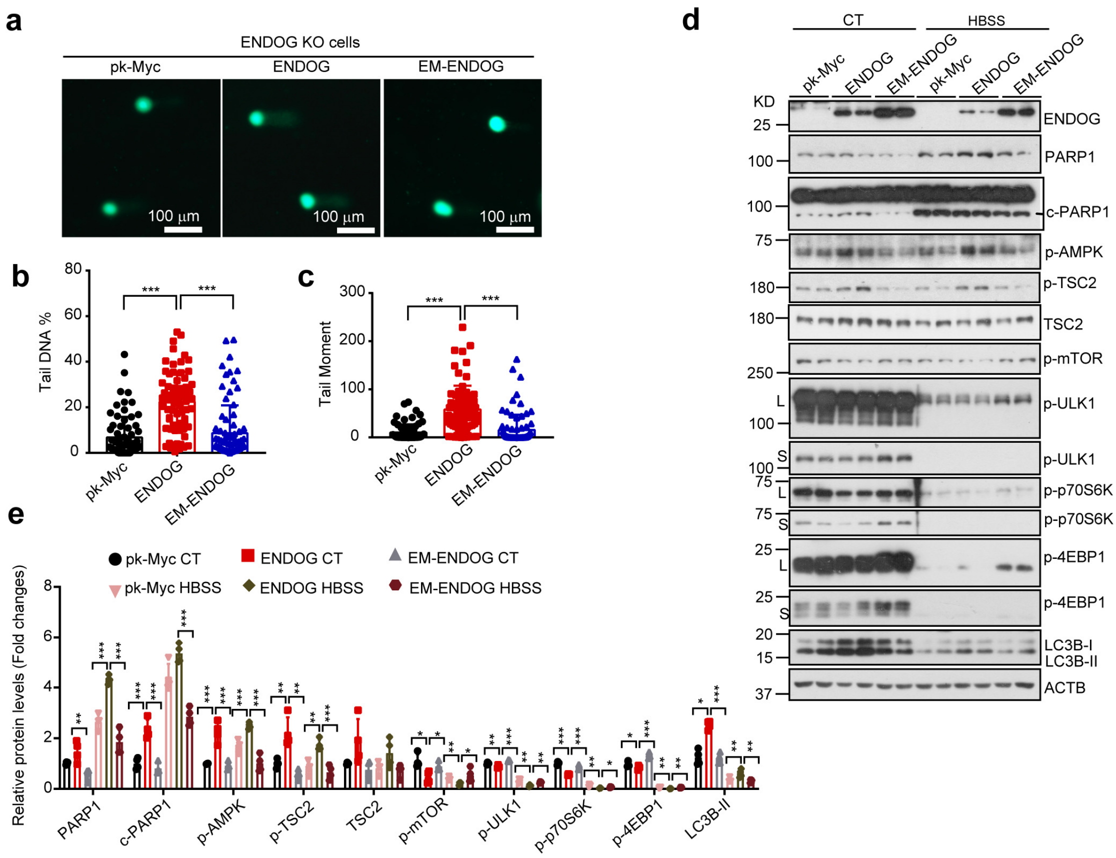

Figure S13. Endonuclease activity of ENDOG is essential for ENDOG-mediated DNA damage and mTOR repression under the starvation. a-c. Representative images of comet assay (**a**) and the quantification of tail DNA (**b**) and tail moment (**c**).

(ENDOG knockout cells were transfected with pK-Myc, wild-type and EM-ENDOG for 48 hours; n = 75- 150 independent cells; data are presented as mean values ± SD; *** $p < 0.0001$). **d-e.** Western blots (**d**) and quantification (**e**) of the indicated proteins. (ENDOG knockout cells were transfected with pK-Myc, wild-type and EM-ENDOG for 48 hours and treated with HBSS for 6 hours; n = 4 independent samples; S: short exposure; L: long exposure; data are presented as mean values ± SD; * $p < 0.05$; ** $p < 0.01$; *** $p < 0.001$). Source data are provided as a Source Data file.

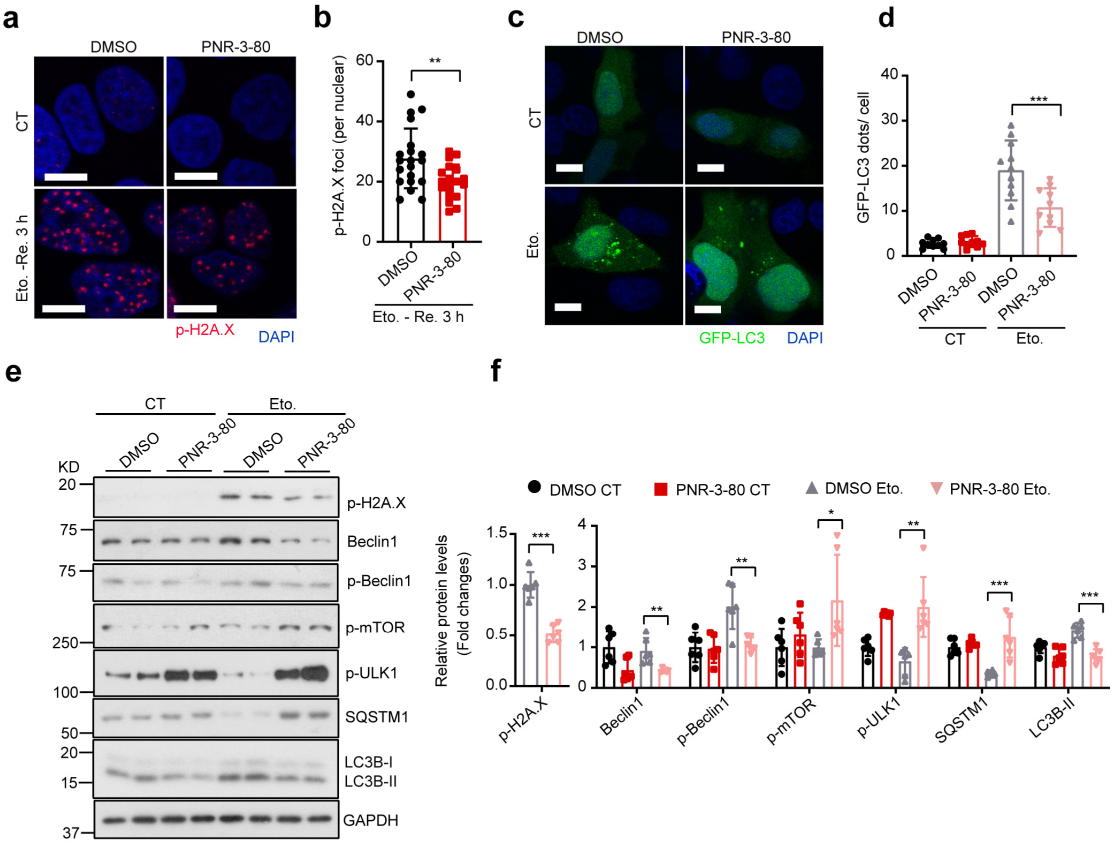

Figure S14. Inhibition of ENDOG activity represses DNA damage-induced autophagy. a-b. Representative images (**a**) and respective quantitative results (**b**) of p-H2A.X foci in L02 cells (Scale bar = 10 μm; n = 100 independent cells exaimined over 3 independent experiments; data are presented as mean values ± SD; ** $p < 0.01$). **c-d.**

Representative images (**c**) and respective quantitative results (**d**) of GFP-LC3 puncta in L02 cells (Scale bar = 10 μm; n = 100 independent cells exaimined over 3 independent experiments; data are presented as mean values ± SD; *** $p < 0.001$). **e-f.** Western blots (**e**) and quantification (**f**) of the indicated proteins (n = 4 independent samples). (PNR-3-80: ENDOG inhibitor, 50 μM for 24 hours; Eto.: etoposide, 50 μM for 1 hour; data are presented as mean values ± SD; * $p < 0.05$; ** $p < 0.01$; *** $p <0.001$). Source data are provided as a Source Data file.

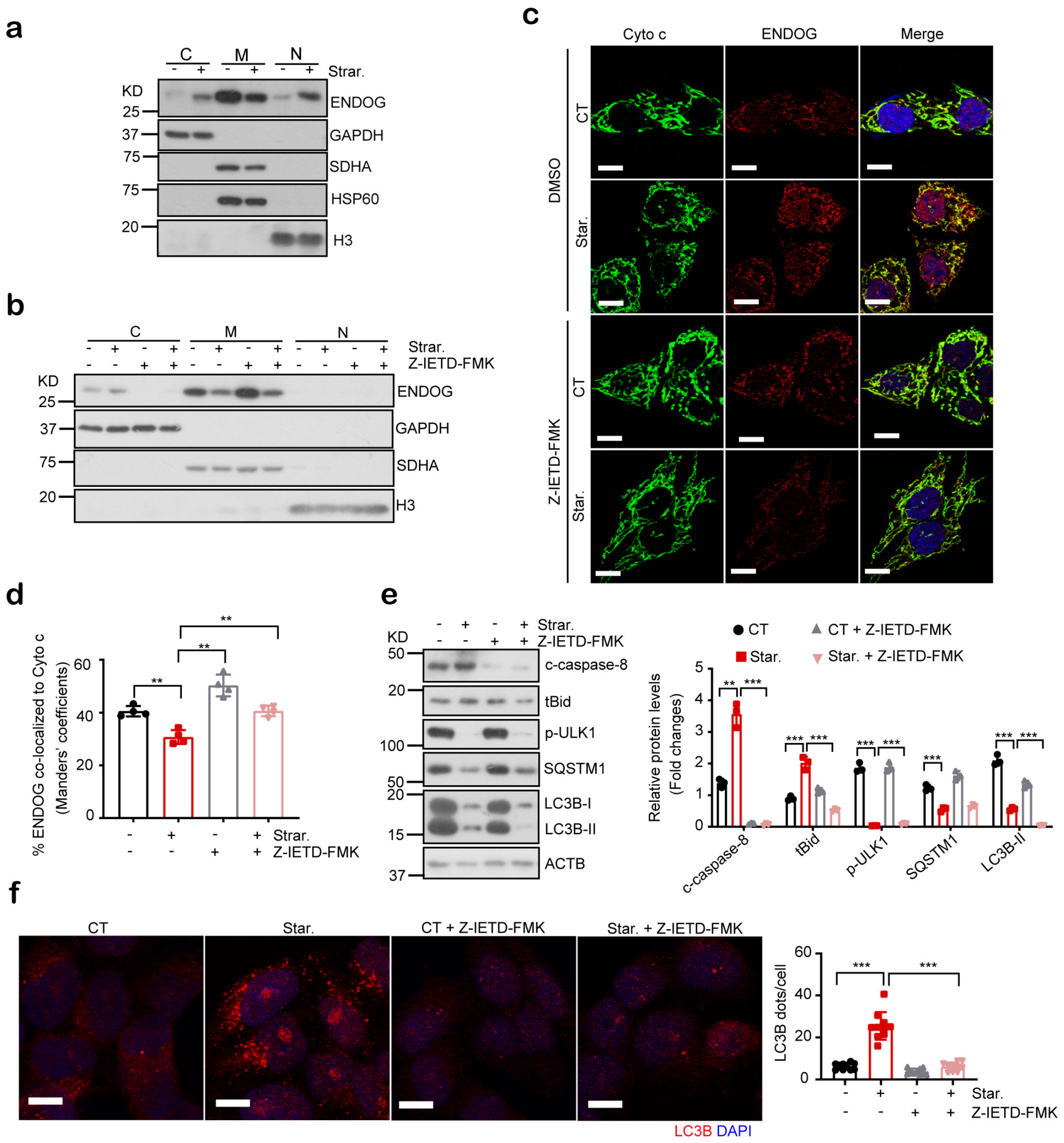

Figure S15. Caspase-8/tBid mediate starvation induced autophagy and the release of ENDOG from mitochondria. a. Sub-cellular fractionation isolation shows starvation promotes the release of ENDOG from mitochondria to cytosol and nuclei in L02 cells (Star.: Starvation for 6 hours; C: cytoplasm; M: mitochondria; N: nuclear biological repeated three times). **b.** Inhibition of caspase-8 represses the release of ENDOG from mitochondria in L02 cells (Star.: Starvation for 6 hours; Z-IETD-FMK: caspase-8 inhibitor, 50 μM for 2 hours; C: cytoplasm; M: mitochondria; N: nuclear; biological repeated three times). **c.** Representativeimmunofluorescent staining images

of Cytochrome c (mitochondria marker) and ENDOG under caspase-8 inhibitor and starvation treatment (Star.: starvation for 6 hours, Z-IETD-FMK: 10 μM for 2 hours; Scale bar =10 μm; biological repeated three times). **d.** Quantitative analysis for thecolocalization of ENDOG with mitochondria (Cyto c) in (**c**) by ImageJ plugin JACOP (n = 4 independent field; data are presented as mean values ± SD, ** $p < 0.01$). **e.** Western blots (left) and quantitative results (right) showed inhibition of caspase-8 represses starvation-induced autophagy (n = 3 independent experiments; data are presented as mean values ± SD). **f.** Immunofluorescent staining of endogenous LC3B (Star. : starvation for 6 hours, Z-IETD-FMK: 10 μM for 2 hours; Scale bar = 10 μm; 100 independent cells exaimined over 3 independent experiments; data are presented as mean values ± SD; *** $p < 0.001$). Source data are provided as a Source Data file.

Table S1. Candidate 14-3-3-binding sites of ENDOG

Position	Peptide	ANN	PSSM	SVM	Consensus
12	AGLTLA[S]GAGL	0.118	-0.242	-1.087	-0.404
77	GLAQLK[S]RESY	0.296	0.020	-0.532	-0.072
80	QLKSRE[S]YVLC	0.236	0.018	-0.860	-0.202
89	LCYDPR[T]RGAL	0.178	-0.014	-0.886	-0.241
120	DFREDD[S]VHAY	0.500	0.229	-0.434	0.098
128	HAYHRA[T]NADY	0.380	0.138	-0.545	-0.009
135	NADYRG[S]GFDR	0.291	0.023	-0.511	-0.066
151	AANHRW[S]QKAM	0.243	-0.065	-0.857	-0.226
158	QKAMDD[T]FYLS	0.269	-0.069	-0.606	-0.135
162	DDTFYL[S]NVAP	0.091	-0.174	-1.274	-0.452
183	NNLEKY[S]RSLT	0.039	-0.304	-1.443	-0.569
185	LEKYSR[S]LTRS	0.499	0.191	-0.233	0.152
187	KYSRSL[T]RSYQ	0.461	0.766	-0.114	0.371
189	SRSLTR[S]YQNV	0.076	0.117	-1.615	-0.474
197	QNVYVC[T]GPLF	0.394	0.342	-0.252	0.161
205	PLFLPR[T]EADG	0.321	0.091	-0.592	-0.060
211	TEADGK[S]YVKY	0.105	-0.129	-1.133	-0.386
227	NHVAVP[T]HFFK	0.128	-0.251	-1.327	-0.482
246	GQIELR[T]YVMP	0.031	-0.252	-1.869	-0.697

269	FLVPIE[S]IERA	0.157	-0.115	-0.892	-0.283
274	ESIERA[S]GLLF	0.097	-0.015	-1.104	-0.341
288	ILARAG[S]LKAI	0.916	1.139	1.564	1.206
293	GSLKAI[T]AGSK	0.491	0.067	-0.353	0.068
296	KAITAG[S]K	0.160	0.021	-1.083	-0.301

(Data analysis from http://www.compbio.dundee.ac.uk/1433pred)

ANN - Artificial Neural Network (cut-off = 0.55)

PSSM - Position-Specific Scoring Matrix (cut-off = 0.80)

SVM - Support Vector Machine (cut-off = 0.25)

Consensus - Average of the scores provided by the three methods (cut-off = 0.50)

Table S2. Primary antibodies used in the present study.

Antibody	Company (catalognumber)	Application
Parimary antibodies		
ACTB	Sigma (A8481)	WB, 1:10000
ATG5	Cell Signaling Technology (12994)	WB, 1:5000
ATG7	Sigma (A2857)	WB, 1:5000
ENDOG	Cell Signaling Technology (4969)	WB, 1:1000
ENDOG	NOVUS (IMG-5565-2)	IF, 1:100
Flag-tag	Sigma (F1804)	IP, 1:1000
GFP	Sigma (11814460001)	WB, 1:10000
LC3B	Sigma (L7543)	WB, 1:5000
Myc-tag	Proteintech (16286-1-AP)	IP, 1:1000
p-4EBP1 (Thr 37/46)	Cell Signaling Technology (2855)	WB, 1:1000
p-mTOR (Ser 2448)	Cell Signaling Technology (5536)	WB, 1:2000

mTOR	Cell Signaling Technology (2983)	WB,1:2000
p-p70S6K (Thr 389)	Cell Signaling Technology (9234)	WB, 1:1000
p-ULK1 (Ser 757)	Cell Signaling Technology (14202)	WB, 1:1000
SQSTM1	Sigma (P0067)	WB, 1:10000
Phospho-Ser/Thr	Cell Signaling Technology (25081)	WB, 1:1000
14-3-3γ	Proteintech (12381-1-AP)	WB/IP, 1:1000
p-H2A.X (Ser139)	Cell Signaling Technology (9718)	WB, 1:1000
p-ATM (Ser1981)	Cell Signaling Technology (5883)	WB, 1:1000
p-p53 (Ser15)	Cell Signaling Technology (9286)	WB, 1:1000
p-CHK1	Cell Signaling Technology (2348)	WB, 1:2000
p-CHK2	Cell Signaling Technology (2197)	WB, 1:2000
TSC2	Proteintech (20004-1-AP)	WB, 1:1000
Vps34	Proteintech (12452-1-AP)	WB, 1:2000
GSK-3β	Proteintech(22104-1-AP)	WB, 1:5000
Phospho-Ser/Thr	Cell Signaling Technology (25081)	WB, 1:1000
Beclin1	Cell Signaling Technology (3495)	WB, 1:1000
p-Beclin1 (Ser 93)	Cell Signaling Technology (14717)	WB, 1:1000
ATG13	Cell Signaling Technology (13273)	WB, 1:5000
p-ATG13 (Ser 355)	Cell Signaling Technology (26839)	WB, 1:1000
ATG14	Cell Signaling Technology (96752)	WB, 1:5000
p-ATG14 (Ser 29)	Cell Signaling Technology (92340)	WB, 1:1000
ATG12	Cell Signaling Technology (4180)	WB, 1:5000
p-TSC2 (Ser 1387)	Cell Signaling Technology (5584)	WB, 1:1000
PARP-1	Cell Signaling Technology (9532)	WB, 1:100
p-AMPK (Thr 172)	Cell Signaling Technology (50081)	WB, 1:1000
DNA-PK	Proteintech (19983-1-AP)	WB, 1:1000
p-ATR (Ser 428)	Cell Signaling Technology (2853)	WB, 1:1000
SDHA	Proteintech (14865-1-AP）	WB, 1:5000
Hsp60	Proteintech (15282-1-AP)	WB, 1:5000

H3	Cell Signaling Technology (9715)	WB, 1:5000
Caspase-8	Cell Signaling Technology (9746)	WB, 1:10000
Bid	Cell Signaling Technology (20023)	WB, 1:1000
Cytochrome c	Santa cruz biotechnology (13561)	WB, 1:1000
Secondary antibodies		
Peroxidase AffiniPure Goat Anti-Rabbit IgG	Jackson ImmunoResearch (111-035-144)	WB, 1:5000-1:10000
Peroxidase AffiniPure Goat Anti-Mouse IgG	Jackson ImmunoResearch (115-035-146)	WB, 1:5000-1:10000
Alexa Fluor® 594-AffiniPure goat anti-rabbit	Jackson ImmunoResearch (115-585-146)	IF, 1:200-400
Alexa Fluor® 488-AffiniPure goat anti-mouse IgG	Jackson ImmunoResearch (115-545-146)	IF, 1:200-400
Mouse Anti-Rabbit IgG (Light-Chain Specific) (D4W3E)	Cell Signaling Technology (93702)	IP, 1:1000
VeriBlot for IP Detection Reagent (HRP)	Abcam (ab131366)	IP, 1:1000

Table S3. Sequences of primers used in the present study

Gene names	Sequences, 5’ to 3’
Human-*ATG3*	TTCAGTTCACCCATGCAGGC

	GTTAACAGCCATTTTGCCACTAA
Human-*ATG5*	GGGTCCCTCTTGGGGTACAT
	ACCACACATCTCGAAGCACA
Human-*ATG7*	GAGCAGCCTTGTGAGAGACA
	GGATGCACTGGATACCAGCA
Human-*ATG12*	CGAACACGAACCATCCAAGG
	TGGTCTGGGGAAGGAGCAAA
Human-*TFEB*	TTCCAACAAGGGAAGGTGACAT
	TGGCTCCCAGCCTGAGC
Human-*BDR4*	GTTGATGTGATTGCCGGCTC
	GTGCAGAAAGCTGTTTCGGA
Human-*Zkscan3*	GGTAGAGGGCCGTTACCGAG
	TATGTGACCCATGCATCCCG
Human-*ENDOG*	GGTCAAGCTGCGGCTATATT
	CGACACGTTCTACCTGAGCA
Human-*ACTB*	GTTGTCGACGACGAGCG
	GCACAGAGCCTCGCCTT
