# Supplementary material for: Endonuclease G promotes autophagy by suppressing mTOR signaling and activating the DNA damage response
Source: Nat Commun. 2021 Jan 20;12:476. doi: 10.1038/s41467-020-20780-2 (PMC7817833; doi:10.1038/s41467-020-20780-2)
Supplement: Supplementary file 3 — Reporting Summary [file 41467_2020_20780_MOESM3_ESM.pdf]

nature research

Corresponding author(s): Qinghua Zhou
Last updated by author(s): Dec 13, 2020

Reporting Summary

Nature Research wishes to improve the reproducibility of the work that we publish. This form provides structure for consistency and transparency in reporting. For further information on Nature Research policies, see our Editorial Policies and the Editorial Policy Checklist.

Statistics

For all statistical analyses, confirm that the following items are present in the figure legend, table legend, main text, or Methods section.

n/a	Confirmed	
☐	☒	The exact sample size (*n*) for each experimental group/condition, given as a discrete number and unit of measurement
☐	☒	A statement on whether measurements were taken from distinct samples or whether the same sample was measured repeatedly
☐	☒	The statistical test(s) used AND whether they are one- or two-sided<br>*Only common tests should be described solely by name; describe more complex techniques in the Methods section.*
☒	☐	A description of all covariates tested
☒	☐	A description of any assumptions or corrections, such as tests of normality and adjustment for multiple comparisons
☐	☒	A full description of the statistical parameters including central tendency (e.g. means) or other basic estimates (e.g. regression coefficient) AND variation (e.g. standard deviation) or associated estimates of uncertainty (e.g. confidence intervals)
☐	☒	For null hypothesis testing, the test statistic (e.g. *F*, *t*, *r*) with confidence intervals, effect sizes, degrees of freedom and *P* value noted<br>*Give P values as exact values whenever suitable.*
☒	☐	For Bayesian analysis, information on the choice of priors and Markov chain Monte Carlo settings
☒	☐	For hierarchical and complex designs, identification of the appropriate level for tests and full reporting of outcomes
☒	☐	Estimates of effect sizes (e.g. Cohen's *d*, Pearson's *r*), indicating how they were calculated

Our web collection on statistics for biologists contains articles on many of the points above.

Software and code

Policy information about availability of computer code

Data collection	Leica TCS SP8 for confocal images, CFX Manager™ Software for q-PCR, Quantity One 1-D for western blots, COMET Assay Software Project (CASP software) for comet assay
Data analysis	GraphPad Prism 8.0, Adobe Illustrator CC 2014 Adobe Photoshop CS5, Image J , Quantity One 4.6.3, COMET Assay Software Project (CASP-1.2.3.b1) , Image-Pro-Plus 6.0 software

For manuscripts utilizing custom algorithms or software that are central to the research but not yet described in published literature, software must be made available to editors and reviewers. We strongly encourage code deposition in a community repository (e.g. GitHub). See the Nature Research guidelines for submitting code & software for further information.

Data

Policy information about availability of data

All manuscripts must include a data availability statement. This statement should provide the following information, where applicable:

- Accession codes, unique identifiers, or web links for publicly available datasets
- A list of figures that have associated raw data
- A description of any restrictions on data availability

The source data are provided with this paper, all relevant data are available from the corresponding author upon any reasonable request.

Field-specific reporting

Please select the one below that is the best fit for your research. If you are not sure, read the appropriate sections before making your selection.

☒ Life sciences ☐ Behavioural & social sciences ☐ Ecological, evolutionary & environmental sciences

For a reference copy of the document with all sections, see nature.com/documents/nr-reporting-summary-flat.pdf

Life sciences study design

All studies must disclose on these points even when the disclosure is negative.

Sample size	All data presented represent the mean ± s.d. of at least three biological replicates or three independent experiments. Difference between three or more means was assessed using One-way ANOVA ,Difference between two means was assessed by an Unpaired Two-tailed student's t-test in GraphPad Prism version 8.0
Data exclusions	No data were excluded in our study
Replication	three or more independent experiments were performed or sufficient sample sizes were involved.
Randomization	The samples are randomly grouped base on the genotype, Mice analyzed were litter mates, age and sex-matched.
Blinding	For the GFP-LC3 or mCherry-GFP-LC3 puncta, the p-H2A.X foci analysis and comet assay experiments, the investigator were blinded to group allocation. For other experiments, such as WB, q-PCR .etc, the Investigators were not blinded during experiments,as the same investigator performed those experiments and analyzed the data.

Reporting for specific materials, systems and methods

We require information from authors about some types of materials, experimental systems and methods used in many studies. Here, indicate whether each material, system or method listed is relevant to your study. If you are not sure if a list item applies to your research, read the appropriate section before selecting a response.

Materials & experimental systems

n/a	Involved in the study
☐	☒ Antibodies
☐	☒ Eukaryotic cell lines
☒	☐ Palaeontology and archaeology
☐	☒ Animals and other organisms
☒	☐ Human research participants
☒	☐ Clinical data
☒	☐ Dual use research of concern

Methods

n/a	Involved in the study
☒	☐ ChIP-seq
☒	☐ Flow cytometry
☒	☐ MRI-based neuroimaging

Antibodies

Antibodies used

Primary antibodies:
ENDOG (Cell Signaling Technology, Cat: 4969, Lot: 2, for western blot, 1:1000);
ENDOG (NOVUS, Cat: IMG-5565-2, Lot: 3035-1901, for immunofluorescent staining, 1:100);
SQSTM1 (Sigma, Cat: P0067, for western blot, 1:10000);
LC3B (Sigma, Cat: L7543, Lot: 048M4810V, for western blot, 1:5000);
ACTB (Sigma, Cat: A8481; Clone:2-2.1.14.17,for western blot, 1:10000);
GFP (Roche; Cat: 11814460001; Clone: 7.1 and 13.1; Lot: 27575600; for western blot, 1:10000);
p-mTOR (Cell Signaling Technology, Cat: 5536T, Clone: D9C2, Lot: 2, for western blot,1:2000);
p-ULK1 (Cell Signaling Technology, Cat: 14202, Clone: D706U, Lot: 2, for for western blot,1:2000);
p-p70S6K (Cell Signaling Technology, Cat: 9234T, Clone: 108D2, Lot: 12, for western blot,1:1000);
p-4EBP-1 (Cell Signaling Technology, Cat:2855T, Clone: 236B4, Lot: 23, for western blot, 1:1000);
mTOR (Cell Signaling Technology, Cat:2983T, Clone: 7C10, Lot: 16, for western blot,1:2000);
TSC2 (Proteintech, Cat: 20004-1-AP, Lot: 00057391, for western blot,1:1000);
Vps34 (Proteintech, Cat: 12452-1-AP, Lot: 00058079, for western blot,1:2000);
14-3-3 (Proteintech, Cat: 12381-1-AP, Lot: 00025589, for western blot and immunoprecipitation, 1:5000/1:1000);
Myc-tag (Proteintech, Cat: 60003-2-Ig, Lot: 10003656, for immunoprecipitation, 1:1000);
Phospho-Ser/Thr Motif [pS/T] (Cell Signaling Technology, Cat: 25081, for western blot,1:1000);
GSK-3b (Proteintech, Cat: 22104-1-AP, Lot: 00058215, for western blot,1:5000);
p-ATM (Cell Signaling Technology, Cat: 5883T; Clone: D6H9; Lot: 6, for western blot,1:1000);
p-p53 (Cell Signaling Technology, Cat: 9286, Clone: 16G8, Lot: 15, for western blot,1:1000);
p-CHK1 (Cell Signaling Technology, Cat: 2348T, Clone: 133D3, Lot: 18, for western blot,1:2000);

April 2020

p-CHK2 (Cell Signaling Technology, Cat: 2197T, Clone: C13C1, Lot: 12, for western blot,1:2000);
p-H2A.X (Cell Signaling Technology, Cat: 9718, Clone: 20E3, Lot: 17, for western blot, 1:1000);
GAPDH (Cell Signaling Technology, Cat:2118S, Clone: 14C10, Lot: 14, for western blot,1:10000);
SDHA (Proteintech, Cat: 14865-1-AP, Lot: 00050301, for western blot, 1:5000);
Hsp60 (Proteintech, Cat: 15282-1-AP, Lot: 00025207, for western blot, 1:5000);
H3 (Cell Signaling Technology, Cat:9715S, , Lot:20, for western blot,1:10000);
Caspase-8 (Cell Signaling Technology, Cat: 9746, Clone: 1C12, Lot: 20, for western blot, 1:1000);
Bid (Cell Signaling Technology, Cat: 20023, Lot: 5, for western blot,1:1000);
Cytochrome c (Santa cruz biotechnology, Cat: sc-13561, Clone: 6H2, Lot: 130414, for immunofluorescent staining, 1:200);
Becn1 (Cell Signaling Technology, Cat: 3495, Lot: 6, for western blot,1:1000);
p-Becn1 (Cell Signaling Technology, Cat: 14717, Lot: 1, for western blot,1:2000);
ATG13 (Cell Signaling Technology, Cat: 13273, Lot: 3, for western blot,1:2000);
p-ATG13 (Cell Signaling Technology, Cat: 26839, Lot: 1, for western blot,1:2000);
ATG14 (Cell Signaling Technology, Cat: 96752, Lot: 1, for western blot,1:2000);
p-ATG14 (Cell Signaling Technology, Cat: 92340, Lot: 1, for western blot,1:2000);
ATG5 (Cell Signaling Technology, Cat: 12994, Lot: 41, for western blot,1:2000);
ATG7 (Sigma, Cat: A2856, Lot: 076M4824V, for western blot,1:2000);
ATG12 (Cell Signaling Technology, Cat: 4180, Lot: 3, for western blot,1:2000);
p-TSC2 (Cell Signaling Technology, Cat: 3390, Lot: 2, for western blot,1:2000);
PARP-1 (Cell Signaling Technology, Cat: 9532, Lot: 9, for western blot,1:2000);
p-AMPK (Cell Signaling Technology, Cat: 50081, Lot: 3, for western blot,1:2000);
DNA-PK (Proteintech, Cat: 19983-1-AP, Lot: 00042867, for western blot,1:2000);
p-ATR (Cell Signaling Technology, Cat: 2853, Lot: 9, for western blot,1:2000).

Secondary antibodies:
Peroxidase AffiniPure Goat Anti-Rabbit IgG (Jackson,111-035-144, for western blot,1:5000-1:10000)
Peroxidase AffiniPure Goat Anti-Mouse IgG(Jackson, 115-035-146, for western blot,1:5000-1:10000)
Alexa Fluor® 594-AffiniPure goat anti-rabbit (Jackson, 115-585-146, for IF, 1:200-400)
Alexa Fluor® 488-AffiniPure goat anti-mouse IgG (Jackson, 115-545-146, for IF, 1:200-400)
Mouse Anti-Rabbit IgG (Light-Chain Specific) (D4W3E) (Cell Signaling Technology, Cat:93702 , for IP 1:1000)
VeriBlot for IP Detection Reagent (HRP) (Abcam,ab131366, for IP 1:1000)

Validation

All commercial antibodies were validated by the manufacturer for the species and application used in this study. We used antibodies recommended by the manufacturer for the species and application.

Eukaryotic cell lines

Policy information about cell lines

Cell line source(s)

L02(HL-7702) and 293T cell were purchased from the Shanghai Cell Bank, Type Culture Collection Committee, Chinese Academy of Sciences. Hepatoma cells (HepG2, MHCC97-H and PLC/PRF/5, gifts from Dr. Liang Chen, Jinan University), which also purchased from ATCC.

Authentication

Non of the cell lines used were authenticated by ourself.

Mycoplasma contamination

All cell lines used in the study were regularly tested for mycoplasma contamination and were determined to be negative.

Commonly misidentified lines
(See ICLAC register)

No misidentified lines were used.

Animals and other organisms

Policy information about studies involving animals; ARRIVE guidelines recommended for reporting animal research

Laboratory animals

Endog knockout mice were generated by Cyagen Biosciences (Guangzhou) Inc, and F0 mice were mated with the wild-type mice (C57/B6) to generate the F1 mice (Endog+/-); F1 male mice were crossed with F1 female mice to generate the F2 mice (Endog+/- and Endog-/-) for experiments. Male mice at age 3-month old were used for the study. Mice were maintained on a 12 light / 12 dark cycle (light on 7 AM to 7 PM), at a room temperature of 22°C ± 2°C, humidity of 50% ± 5%, with free access to food and water.

C. elegans, cps-6 (tm3222) was hybridized with the GFP::LGG-1 strain. PCR genotyping was used to verify the cps-6; GFP::LGG-1 strains. For autophagy analysis, the GFP::LGG-1 and cps-6; GFP::LGG-1 strains were starved for 4 hours. Following the starvation treatment, GFP::LGG-1 puncta in seam cells and pharyngeal muscle were imaged and counted.

Drosophila second-instarlarvae were collected 72-96 hours after the eggs were laid and cultured in vials containing 20% sucrose (starvation conditions) for 4 hours.

Wild animals

The study did not involve wild animals

Field-collected samples

The study did not involve samples collected from the field

Ethics oversight

The animals were handled according to the Guidelines of the China Animal Welfare Legislation and as approved by the Laboratory Animal Ethics Committee of Jinan University (Guangzhou).

Note that full information on the approval of the study protocol must also be provided in the manuscript.
